# Editorial: The present and future of chrono-nutrition studies

**DOI:** 10.3389/fnut.2023.1183320

**Published:** 2023-04-03

**Authors:** Yu Tahara, Jingyi Qian, Hideaki Oike, Carolina Escobar

**Affiliations:** ^1^Graduate School of Biomedical and Health Sciences, Hiroshima University, Hiroshima, Japan; ^2^Division of Sleep and Circadian Disorders, Departments of Medicine and Neurology, Brigham and Women's Hospital, Boston, MA, United States; ^3^Division of Sleep Medicine, Harvard Medical School, Boston, MA, United States; ^4^Institute of Livestock and Grassland Science, National Agriculture and Food Research Organization, Tsukuba, Japan; ^5^Departamento de Anatomía, Facultad de Medicina, Universidad Nacional Autónoma de México, Mexico City, Mexico

**Keywords:** circadian clock, eating behavior, obesity, blood pressure, binge eating

## Introduction

The circadian clock plays an important role in the regulation of eating pattern and food absorption/metabolism in mammals. Moreover, nutrient intake modifies the circadian clocks. Such mutual relationship suggests that the timing of food/nutrients intake can act as a key modifiable lifestyle factor for circadian physiology and metabolic health, which has remained to be an active area of investigation in the field of nutrition research. Therefore, “chrono-nutrition” was newly established as an emerging discipline that considers the calorie intake and dietary composition from the perspective of chronobiology. This Research Topic is aimed at collecting papers to understand the recent progress and evidence of chrono-nutrition studies, and to facilitate the development of the global communities of chrono-nutrition research. We appreciated colleagues who submitted manuscripts to this topic, and finally 14 papers were accepted. In this editorial, we summarize those papers and discuss the present and future of the chrono-nutritional research.

## Effects of dietary composition on the circadian system

High-fat diet (HFD) induces obesity and is also known to dampen locomotor activity rhythm and gene expression rhythms in the peripheral tissues in mice. To investigate how non-diet induced obesity affects circadian thermoregulation, Herrera-García et al. showed that *Neotomodon alstoni* mouse, naturally obese mouse, develop low amplitude in their daily rhythms of body temperature, of caloric expenditure, and of cold response, suggesting lower aerobic metabolism and thermoregulation. Importantly, functional foods targeting the clock may help to improve circadian function. For instance, caffeine has a potent effect on the circadian clock by changing the period, phase, and amplitude of its rhythmicity (1). Hironao et al. showed that HFD-induced obesity and circadian clock disturbance could be prevented by the black soybean seed coat polyphenol. Circadian clock disturbances are also common in neurodegenerative disorders. In a mouse model of Huntington's disease, Whittaker et al. demonstrated that ketogenic diet feeding prevented the age-related disturbances of sleep/wake cycle and motor function.

## Time-restricted feeding/eating as a confirmed chrono-nutritional treatment

Time-restricted feeding (TRF, one of intermittent fasting) protocol focus on the best interval/window for food intake. TRF is suggested as an efficient strategy to prevent HFD-induced obesity in mice. In humans, meta-analyses has confirmed that time-restricted eating (TRE) by restricting eating time for 8–10 h in a day (2) can control weight and improve metabolic dysfunctions in those who are overweight or obese. Daytime TRE enhances not only mitochondrial function but also day-night rhythmicity of clock gene expressions (3). Circadian clock entrainment induced by TRE and the consequent behavioral changes were revised by Trzeciak and Steel, they summarized the inconsistency of experimental protocols and suggest a series of future food entrainment studies. The strength of TRE and intermittent fasting was evidenced by da Costa Oliveira et al., they show that intermittent fasting improves hypothalamic signaling of leptin, insulin, autophagy, inflammation, and brain-microbiota axis, contributing to weight loss in obesity. TRE is also effective on the blood pressure in humans by meta-analysis (4). Hou et al. demonstrated that TRE in the light phase resulted in reverse dipping of blood pressure in wild-type and diabetic *db/db* mice.

In spite of the benefits of TRE as compared with other intermittent fasting protocols, the adherence of TRE intervention needs to be improved in the future. O'Neal et al. discuss social, family, work, and other lifestyle factors, that hamper the adherence to TRE, especially with short eating windows of 6–10 h. Although in the basic concept of TRE intervention, participants can consume food anytime during the eating window, Guerrero-Vargas et al. demonstrated that distributed food timing during the TRF (i.e., food given every 3 h in 12-h TRF) in rats had more improving effect on the constant light-induced disturbances of locomotor activity rhythm, body temperature rhythm, and estrus cycle, compared with TRF with free access of food during the 12-h window. Taking together, TRE has been identified as a chrono-nutritional intervention protocol for improving health, such as body weight and blood pressure, with sufficient evidence.

## Timing of nutrient intake

For decades, how much and what we eat are considered most essential modifiable nutritional factors affecting our wellbeings. However, chrono-nutrition studies are pointing out another important pillar of this paradigm: when we eat. Indeed, meta-analyses have demonstrated that breakfast skipping, and bigger dinner are associated with weight gain (5, 6), indicating that late calorie intake increases the risk for obesity. In this issue, Begemann and Oster investigated how late consumption of one of our favorite snacks—chocolate affects metabolism. They found that rest-phase chocolate consumption in mice leads to a higher body temperature and increased locomotor activity compared to active-phase chocolate consumption, which may contribute to the late-night snack-induced weight gain. Cross-sectional study by Imamura et al. showed that sodium/potassium intake at lunch or lipid intake at dinner influenced the blood pressure, suggesting the importance of timing of nutrient intake for our health. Kim et al. demonstrated that protein intake for breakfast in elderly participants, relative to the daily total protein intake, resulted in higher handgrip strength and muscle mass. An experimental study also concluded that milk protein in the morning rather than in the evening favored an increase of muscle mass in elderly women. The authors also confirmed the timing effect of protein intake on the muscle synthesis using overloading-induced muscle hypertrophy model mice (7). Caba-Flores et al. reviewed the importance of contents and timing of breast milk supplementation to the babies, since the glucocorticoids and melatonin would be the circadian cue for the infant clock. Recent study suggests the importance of the timing of nutrient intake on the gestation length in the pregnant mother (8), thus more research on the chrono-nutrition in pregnancy is needed.

## Binge eating disorder vs. circadian disorder

Patients with eating disorders, such as night-eating syndrome, often express evening chronotype with insomnia and overweight. Individuals with binge eating behavior tend to binge during late hours of the day. Moreover, morning bright light therapy is effective for reducing the binge eating behavior, suggesting that a change of the patient's daily rhythms to a morning chronotype can be a successful treatment strategy. One opinion article (Plano et al.) and one systematic scoping review (Romo-Nava et al.) summarized recent findings about the binge eating disorder with the circadian clock and concluded that the circadian system may play a role in the etiology of binge eating behavior. However, further research is needed for understanding a more detailed connection.

## Future directions

As mentioned above, novel chrono-nutritional evidence was already confirmed by the meta-analysis which might be ready for the clinical translation. The newly developed chrono-nutritional research should be applied to the health guidelines, such as national dietary reference intakes, in each country. Furthermore, dietary suggestions should be tailored to the individual when considering chronotype, age, gender, social background (e.g., shiftwork), and genetic background. It is fortunate that a growing number of researchers are joining this chrono-nutritional research area. In Japan, the Japan Chrono-Nutrition Society (JCNS) established in 2014. Society members include physicians, public health nurses, nutritionists, and food companies in addition to nutrition researchers. Around the world, food companies are elaborating products in which the effective timing of intake is suggested. A good example is Brazil, where different products suggest the beneficial effects for sleep or for the activity performance. However, in Japan, based on the Japanese law, products excepting medicine, cannot suggest to the consumers about the timing of intake, which certainly should be changed in the future. In the US, the Dietary Guidelines Advisory Committee appointed by the Departments of Health and Human Services and Agriculture is considering including recommendations on meal timing into the coming *Dietary Guidelines for Americans, 2025–2030*. This highlights the increasing awareness of chrono-nutrition. In Mexico, a country with high levels of overweight and obesity, a growing interest in chrono-nutrition is expressed by nutritionists and food companies. However, more clinical studies are necessary to provide the scientific evidence and benefits for physiology and behavior. Finally, the present collection raises the need to continue the studies on chrono-nutrition and to organize a worldwide chrono-nutrition conference in the future.

## Author contributions

All authors wrote and contributed to the article and approved the submitted version.

## References

[1] Narishige S Kuwahara M Shinozaki A Okada S Ikeda Y Kamagata M. Effects of caffeine on circadian phase, amplitude and period evaluated in cells in vitro and peripheral organs *in vivo* in Per2::luciferase mice. Br J Pharmacol. (2014) 171:5858–69. 10.1111/bph.12890 25160990 PMC4290722

[2] Moon S Kang J Kim SH Chung HS Kim YJ Yu JM. Beneficial effects of time-restricted eating on metabolic diseases: a systemic review and meta-analysis. Nutrients. (2020) 12:1267. 10.3390/nu12051267 32365676 PMC7284632

[3] Di Francesco A Di Germanio C Bernier M de Cabo R. A time to fast. Science. (2018) 362:770–5. 10.1126/science.aau2095 30442801 PMC8504313

[4] Wang W Wei R Pan Q Guo L. Beneficial effect of time-restricted eating on blood pressure: a systematic meta-analysis and meta-regression analysis. Nutr Metab. (2022) 19:77. 10.1186/s12986-022-00711-2 36348493 PMC9644535

[5] Ma X Chen Q Pu Y Guo M Jiang Z Huang W. Skipping breakfast is associated with overweight and obesity: a systematic review and meta-analysis. Obes Res Clin Pract. (2020) 14:1–8. 10.1016/j.orcp.2019.12.002 31918985

[6] Young IE Poobalan A Steinbeck K O'Connor HT Parker HM. Distribution of energy intake across the day and weight loss: a systematic review and meta-analysis. Obes Rev. (2023) 24:e13537. 10.1111/obr.13537 36530130 PMC10078448

[7] Aoyama S Kim HK Hirooka R Tanaka M Shimoda T Chijiki H. Distribution of dietary protein intake in daily meals influences skeletal muscle hypertrophy via the muscle clock. Cell Rep. (2021) 36:109336. 10.1016/j.celrep.2021.109336 34233179

[8] Loy SL Cheung YB Cai S Colega MT Godfrey KM Chong YS. Maternal night-time eating and sleep duration in relation to length of gestation and preterm birth. Clin Nutr. (2020) 39:1935–42. 10.1016/j.clnu.2019.08.018 31493922

